# Cancer cachexia reduces the efficacy of immune checkpoint inhibitors in cancer patients

**DOI:** 10.18632/aging.205652

**Published:** 2024-03-11

**Authors:** Yean Yu, Li Yan, Tianhui Huang, Zhenfu Wu, Juan Liu

**Affiliations:** 1 Department of Nephrology, Wuhan Third Hospital, Tongren Hospital of Wuhan University, Wuhan, China; 2 Department of Critical Care Medicine, Hubei Provincial Hospital of Traditional Chinese Medicine, Wuhan, China; 3 Department of Traditional Chinese Medicine, Wuhan Third Hospital, Tongren Hospital of Wuhan University, Wuhan, China; 4 Department of Abdominal and Pelvic Medical Oncology, Huangshi Central Hospital, Affiliated Hospital of Hubei Polytechnic University, Huangshi, China; 5 Department of Critical Care Medicine, Affiliated Hospital of Hubei University of Chinese Medicine, Wuhan, China

**Keywords:** cachexia, immune checkpoint inhibitors, cancers, non-small cell lung cancer

## Abstract

Objective: Cachexia, a multifactorial syndrome, is frequently noticed in cancer patients. A recent study has shown inconsistent findings about the relationship between cachexia and the efficiency of immune checkpoint inhibitors (ICIs). To analyze this disparity, we did a meta-analysis.

Methods: From the beginning of each database to July 2023, literature describing the association between cachexia and prognosis of ICI-treated patients with solid malignancies was systematically searched in three online databases. Estimates were pooled, and 95% confidence intervals (CIs) were generated.

Results: We analyzed a total of 12 articles, which included data from 1407 patients. The combined results of our analysis showed that cancer patients with cachexia had significantly worse overall survival (HR = 1.88, 95% CI: 1.59–2.22, *p* < 0.001), progression-free survival (HR = 1.84, 95% CI: 1.59–2.12, *p* < 0.001), and time to treatment failure (HR = 2.15, 95% CI: 1.32–3.50, *p* = 0.002). These findings were consistent in both univariate and multivariate analyses. Additionally, while not statistically significant, we observed a trend towards a lower objective response rate in cancer patients with cachexia compared to those without cachexia (OR = 0.59, 95% CI: 0.32–1.09, *p* = 0.093).

Conclusion: Poor survival in cachexia patients suggests a negative relationship between cachexia and ICI efficacy. In clinical practice, the existence of cachexia should be estimated to choose individuals who may benefit from ICIs.

## INTRODUCTION

Immune evasion assumes an important role in the genesis and progression of cancer, constituting one of the foremost hallmarks of cancer [1, 2]. In addition to modulating the immune system, immune checkpoints, which are made up of co-inhibitory and stimulatory signals, can shield cancer cells from immune destruction [1, 3]. Immune checkpoint inhibitors (ICIs), whether administered in isolation or in combination, can yield enduring antitumor effects by strategically reducing the production of negative immunomodulatory factors [4]. The Food and Drug Administration (FDA) has currently approved several ICIs (anti-PD-(L)1 and anti-CTLA-4 antibodies) for use in some cancer indications.

Nonetheless, the efficacy of ICI therapy varies contingent on the specific cancer type, typically spanning from a modest 10% to 40%. Notably, a substantial proportion of patients experience disease progression despite an initial positive response [4, 5]. Conversely, the administration of ICIs carries the risk of potential immune-related adverse events (irAEs), some of which can manifest as severe or even life-threatening [6]. The imperative of identifying, at an early stage, those individuals unlikely to benefit from ICI therapy, thereby averting ineffective interventions and mitigating the risk of severe irAEs, has emerged as a prominent concern in the field of cancer therapy. A diverse array of predictive markers related to the ICI response has been explored, encompassing parameters such as intratumoral PD-L1 expression, tumor mutational burden (TMB), and T-cell infiltration metrics [7]. Nevertheless, devising standardized criteria for the quantification of these markers remains a formidable challenge. Moreover, the complexity of procuring tumor samples before the initiation of therapy poses an additional hurdle. To date, regulatory companion diagnostic approval for ICI treatment has been granted solely for intratumoral PD-L1 detection [8]. Consequently, the quest for novel prognostic markers assumes paramount significance as a means to enhance the clinical outcomes of cancer patients undergoing ICI-based treatment. Cachexia, a condition characterised by progressive functional decline [9], manifests in nearly half of cancer patients [10] and contributes to 20% of cancer-related mortalities [11]. Cachexia is defined as a weight loss of > 5% over 6 months or a weight loss of >2% if the body mass index (BMI) is <20 kg/m^2^ in the absence of simple starvation [12]. Recent studies demonstrated that cachexia is related to reduced effectiveness of chemotherapy, surgery, and targeted treatment in tumor patients [13–15]. It is still unknown how cachexia affects the effectiveness of ICIs in tumor patients. Thus, the objective of our study was to comprehensively estimate the impact of cachexia on ICI-treated cancer patients. The outcomes of this research will contribute to the development of effective treatment strategies that enable precise and cost-effective therapies with minimal adverse effects.

## METHODS

### Search strategy

The preferred reporting items for systematic reviews and meta-analyses criteria were used in this work [16]. From the beginning of each database through July 2023, we searched PubMed, EMBASE, and the Cochrane Library for published papers on the connection between cachexia and ICI efficacy. MeSH terms and keywords such as “Immune Checkpoint Inhibitors (MeSH)”, “PD-1 Inhibitors”, “PD-L1 Inhibitors”, “CTLA-4 Inhibitors”, “Cachexia” (MeSH), and “Weight Loss” (MeSH) were utilised, among others. The detailed search strategy is outlined in Supplementary File 1. Additionally, the grey literature was looked into using Google Scholar, as well as the reference lists of eligible research were manually evaluated. The search results were uploaded to Endnote 20, which allows for the automated removal of duplicates as well as the manual screening of abstracts and full-text publications.

### Study selection

The following criteria were met by articles in English to be included in this study: (i) cancer patients who received ICIs; (ii) research evaluating the influence of cachexia before ICI therapy; (iii) outcomes such as overall survival (OS), progression-free survival (PFS), time to treatment failure (TTF), or objective response rate (ORR) were reported. ORR was defined as the percentage of individuals who achieved complete response or partial response as best response to treatment. Besides, the diagnosis of cachexia was based on the criteria established by Fearon et al. [12]. The following were the exclusion requirements: (i) study designs such as animal studies, case reports, and conference abstracts.

### Data extraction

The data extraction mainly focused on the author, year, study design, study period, study region, treatment, type of cancer, sample size, age, male and female patients, definition of cachexia, and outcomes. HRs, ORs, and 95% CIs were primarily extracted from multivariate analyses, otherwise from univariate analyses or Engauge Digitizer to extract from the Kaplan-Meier survival curve. The Newcastle-Ottawa Scale was utilized to evaluate the standard of the included research. We assigned nine points worth of quality-related criteria to the domains of patient selection, study comparability, and study endpoints. Studies with a score ≥6 were deemed to be of high quality. The above process has been independently completed and cross-checked by two authors, with senior authors consulted on any disputes.

### Statistical methods

Pooled analyses were performed using Stata 15.0. Heterogeneity was estimated using Cochran’s *Q* test and I^2^ statistics. A fixed-effect model with the Inverse Variance method was utilized when *p* > 0.1 and I^2^ <50% indicated non-significant heterogeneity; otherwise, the random-effect model with the DerSimonian-Laird method was applied [17, 18]. To investigate publication bias, the funnel plot, Egger tests, and Begg tests were utilized [19, 20]. By separately removing each study, a sensitivity analysis was done to gauge the stability of the conclusions. Subgroup analyses were performed for the Cox model and the kind of cancer.

## RESULTS

### Literature search results

**Figure 1:**
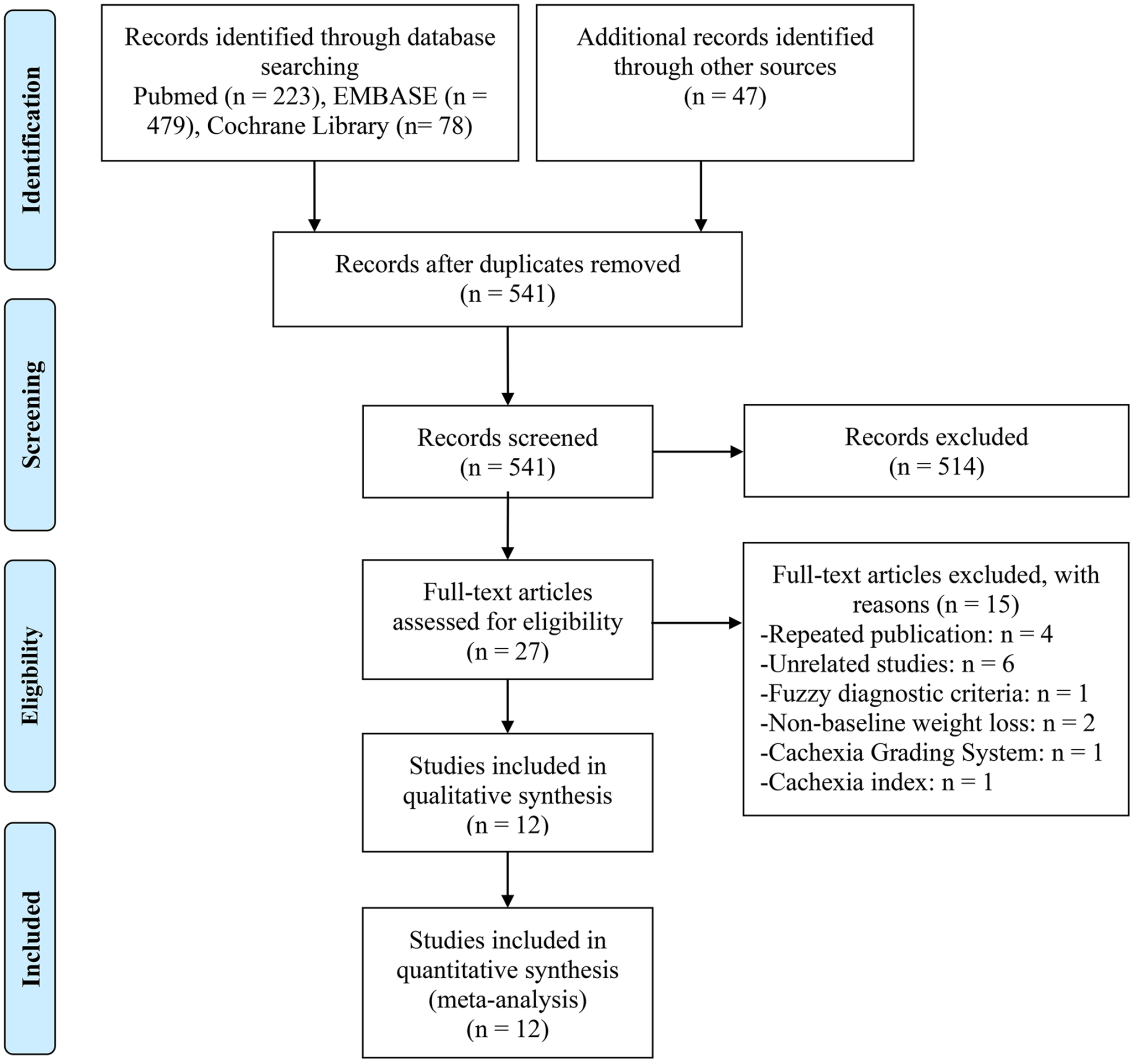
The flow diagram of identifying eligible studies.

827 records were retrieved through the electronic database search for articles (Figure 1). Before screening for titles and abstracts, we eliminated duplicate entries and articles written in languages other than English. After carefully reviewing the full texts of the 27 studies that had been chosen, 12 studies totaling 1407 patients were eventually included in our analysis (Figure 1) [21–32].

Table 1 shows the characteristics of the included studies. Seven studies were performed in Japan, and one study each was conducted in the Netherlands, Italy, Greece, the USA, and France. Ten studies enrolled patients with NSCLC, and one study each enrolled patients with HNC and GC. Ten studies were retrospective, while two studies were prospective. Furthermore, the 12 studies received NOS scores ranging from 6 to 8, underscoring a minimal likelihood of bias (Table 1).

**Table 1:** Main characteristics of the studies included.

**Study**	**Study design**	**Study period**	**Study region**	**ICI treatment**	**Cancer type**	**Sample size**	**Age (years)**	**Sex (male/female)**	**Outcomes**	**NOS**
Willemsen et al. 2023	R	01/2014-03/2020	Netherlands	Anti-CTLA-4 antibodies	HNC	98	63 ± 8.0^a^	83/15	OS	7
Madeddu et al. 2023	P	04/2017-08/2021	Italy	Pembrolizumab, Nivolumab	NSCLC	74	69 ± 11.3^a^	54/20	OS, PFS	7
Matsuo et al. 2023	R	02/2016-10/2020	Japan	Pembrolizumab, Nivolumab, Atezolizumab	NSCLC	183	-	135/48	OS, PFS, ORR	8
Nishioka et al. 2022	R	05/2016-12/2018	Japan	Pembrolizumab, Nivolumab, Atezolizumab	NSCLC	74	68 (33–84)^b^	58/16	ORR	7
Fujii et al. 2022	R	04/2014-06/2020	Japan	Pembrolizumab	NSCLC	53	-	42/11	OS, ORR	6
Jo et al. 2022	R	03/2017-12/2018	Japan	Pembrolizumab	NSCLC	133	-	88/45	OS, PFS, ORR	7
Miyawaki et al. 2022	R	12/2018-12/2020	Japan	Anti-PD-(L)1 antibodies	NSCLC	152	71 (35–88)^b^	113/39	OS, PFS	7
Morimoto et al. 2021	R	01/2019-12/2019	Japan	Pembrolizumab, Bevacizumab, Atezolizumab	NSCLC	196	69 (37–85)^b^	142/54	OS, PFS, ORR	7
Mu et al. 2021	R	06/2011-08/2019	USA	Anti-PD-(L)1 antibodies	NSCLC	175	66 ± 12^a^	96/79	OS, PFS	7
Rounis et al. 2021	P	2017–2020	Greece	Pembrolizumab, Nivolumab, Atezolizumab	NSCLC	83	66 (39–81)^b^	70/13	OS, PFS, ORR	7
Roch et al. 2020	R	07/2015-02/2017	France	Pembrolizumab, Nivolumab	NSCLC	142	64 ± 10.6^a^	93/49	OS, PFS	8
Fujii et al. 2020	R	10/2017-12/2019	Japan	Nivolumab	GC	44	-	23/21	OS, ORR	6

^a^mean ± standard deviation; ^b^medians (ranges). Abbreviations: R: retrospective study; P: prospective study; PD-(L)1: programmed cell death (ligand)-1; CTLA-4: cytotoxic T-lymphocyte-associated protein 4; HNC: head and neck cancer; NSCLC: non-small cell lung cancer; GC: gastric cancer; OS: overall survival; PFS: progression-free survival; ORR: overall response rate.

### Pre-immunotherapy cachexia and OS

**Figure 2:**
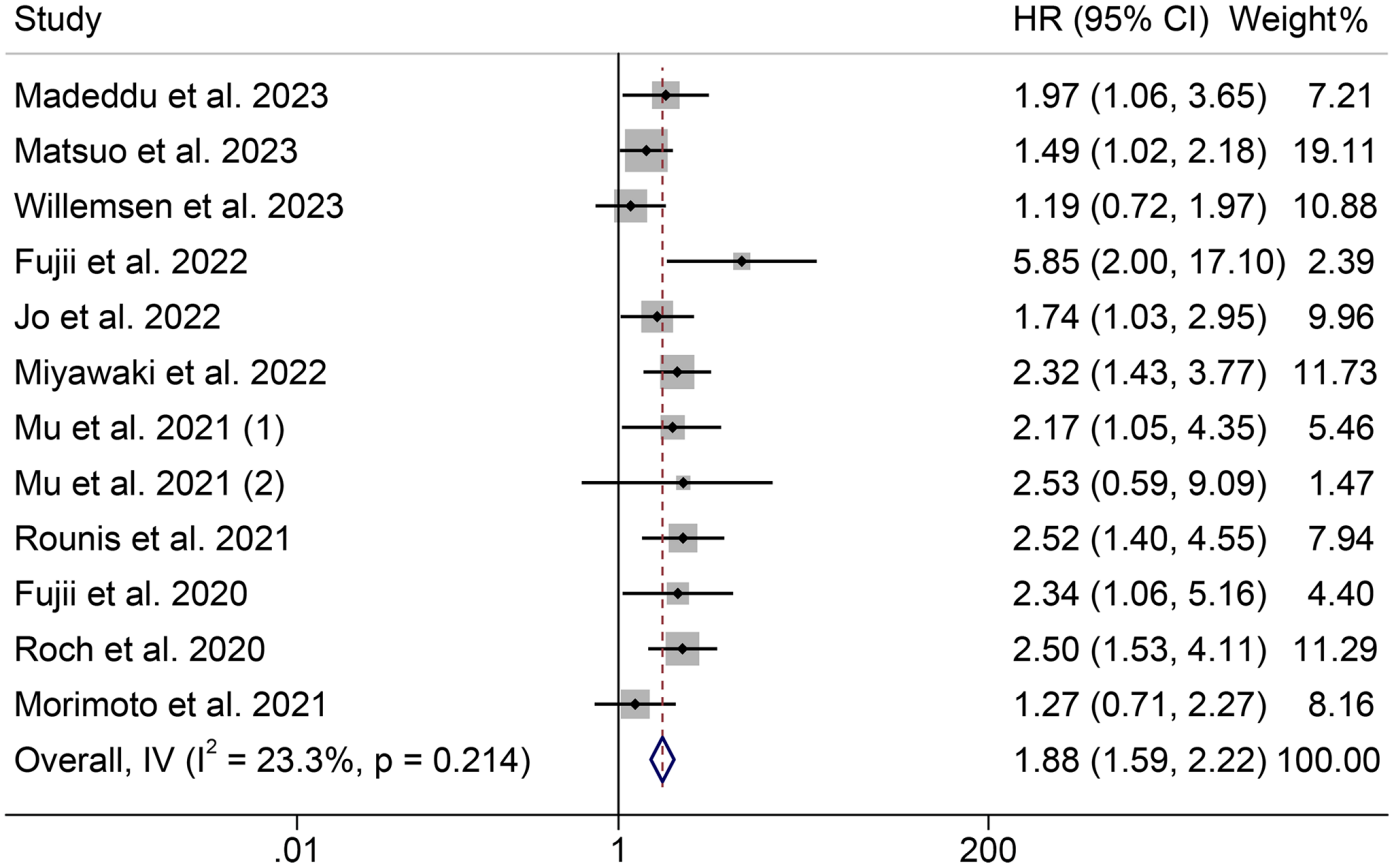
**Forest plots of the relationship between cachexia and overall survival.** Abbreviations: HR: hazard ratio; CI: confidence interval; IV: Inverse Variance method.

The effect of pre-treatment cachexia on OS in cancer patients treated with ICIs was examined in 12 cohorts, including 1333 participants. A fixed-effect model was used since the Cochran *Q* test and I^2^ statistics showed no significant heterogeneity (*p* = 0.214, I^2^ = 23.3%). The pooled results revealed that cancer patients with cachexia had significantly poorer OS than those without cachexia (HR = 1.88, 95% CI: 1.59–2.22, *p* < 0.001, Figure 2). NSCLC patients were recruited in 10 cohorts, and NSCLC patients with cachexia have shorter OS (I^2^ = 15.4%, *p* = 0.301; HR = 1.97, 95% CI: 1.64–2.36, *p* < 0.001, Supplementary Figure 1).

**Figure 3:**
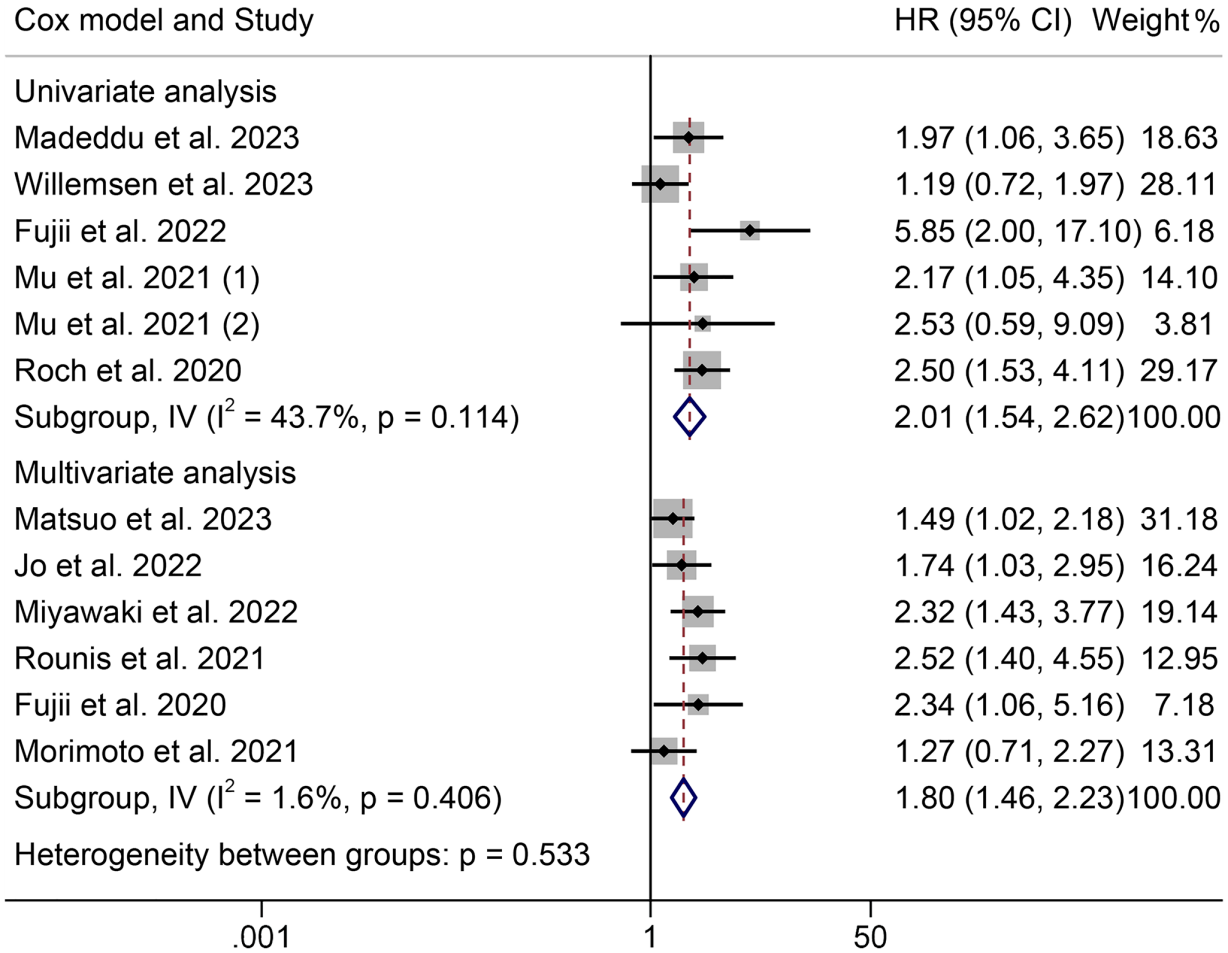
**Forest plots of the association between cachexia and overall survival in the multivariate and univariate analysis.** Abbreviations: HR: hazard ratio; CI: confidence interval; IV: Inverse Variance method.

The HRs for OS according to the Cox proportional hazards model used are shown in Figure 3. Univariate and multivariate analyses were conducted in six cohorts, respectively. The HRs (95% CIs) were 2.01 (1.54–2.62) for univariate analyses and 1.80 (1.46–2.23) for multivariate analyses. There were no significant differences among the different models (*P* = 0.533).

### Pre-treatment cachexia and PFS/TTF

**Figure 4:**
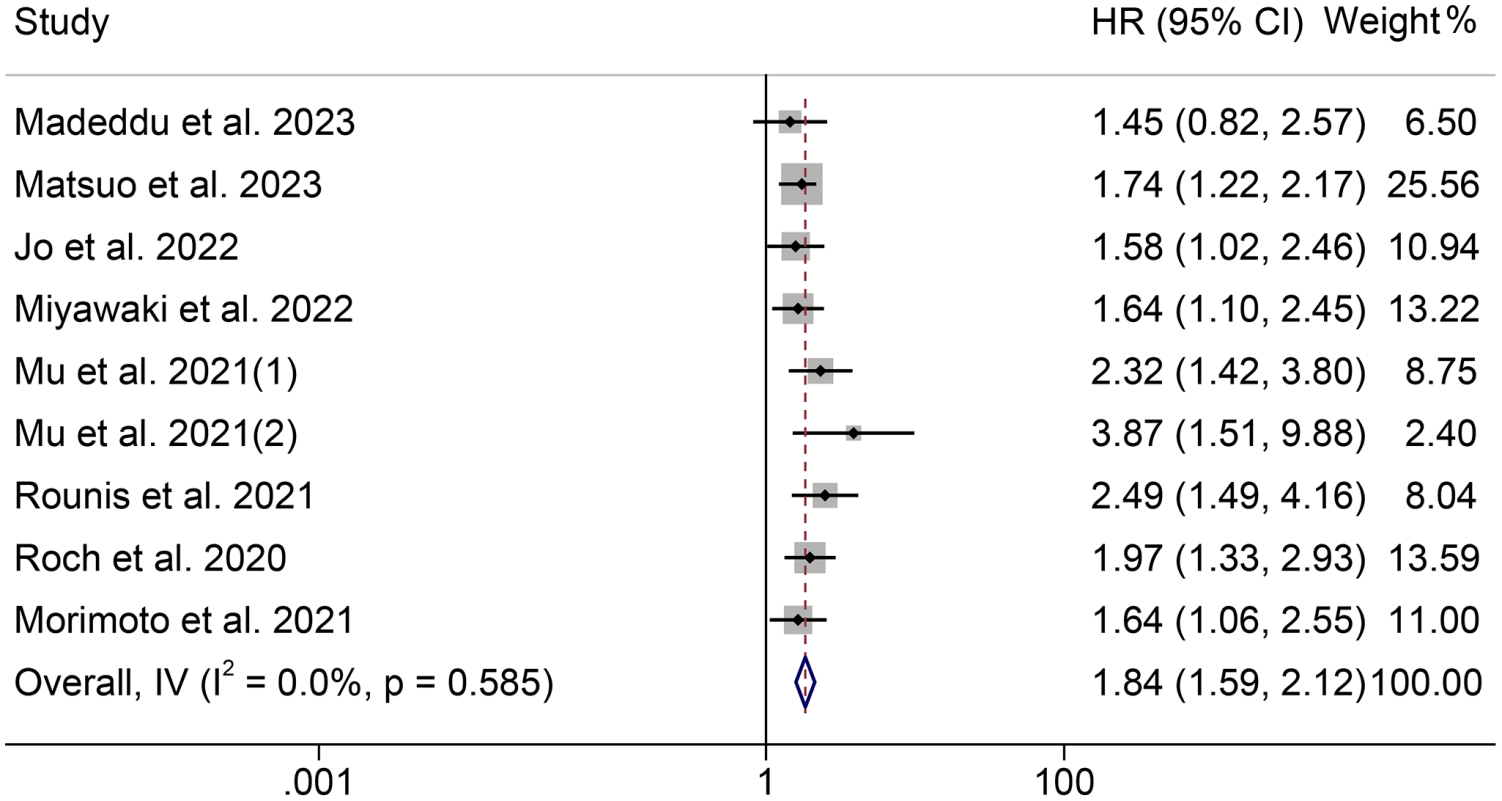
**Forest plots of the relationship between cachexia and progression-free survival.** Abbreviations: HR: hazard ratio; CI: confidence interval; IV: Inverse Variance method.

**Figure 5:**
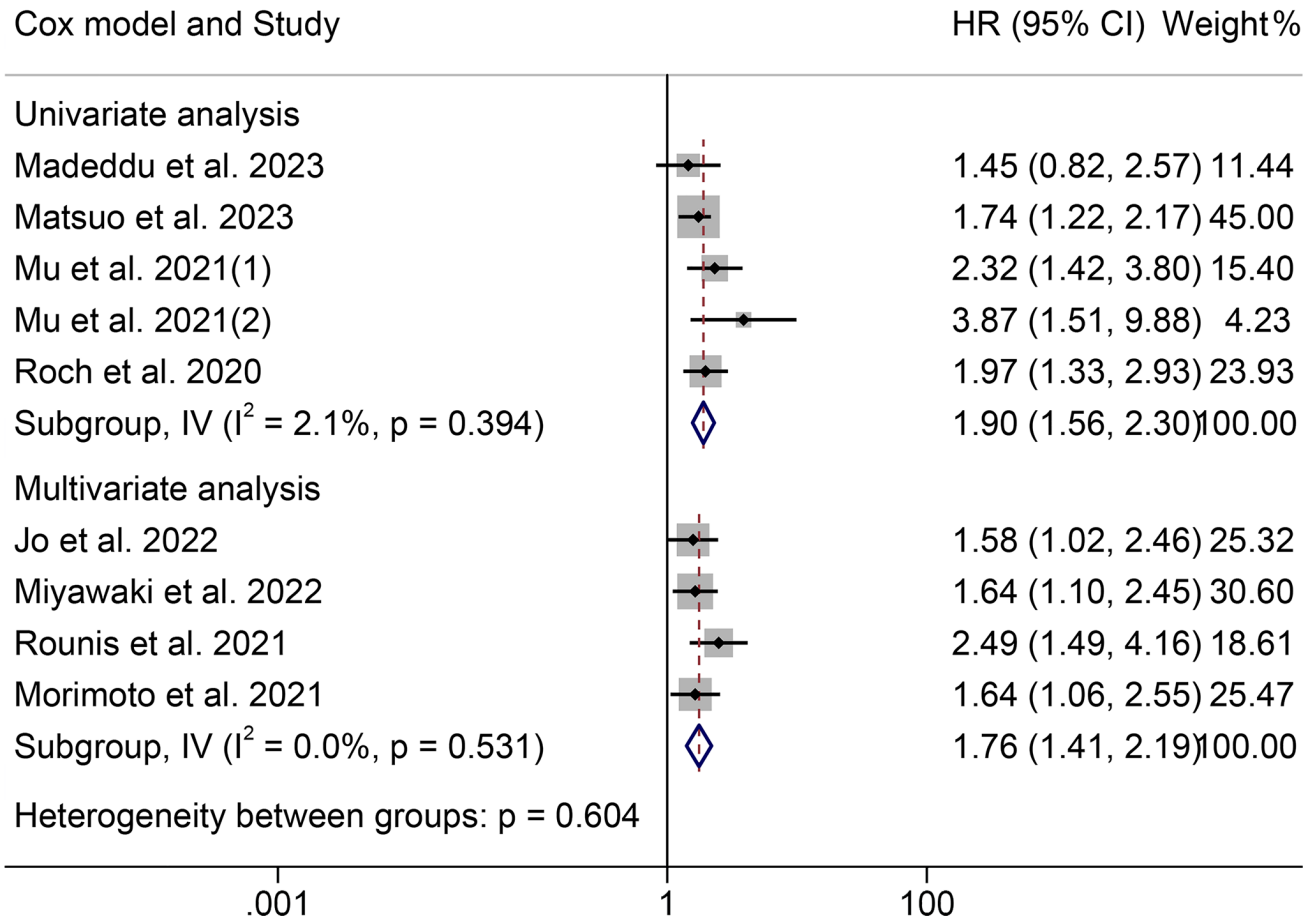
**Forest plots of the association between cachexia and progression-free survival in the multivariate and univariate analysis.** Abbreviations: HR: hazard ratio; CI: confidence interval; IV: Inverse Variance method.

The association between cachexia and PFS was investigated using survival data from nine studies with 1138 participants. Notably, those patients were all diagnosed with NSCLC. As shown in Figure 4, there was no significant heterogeneity among studies (I^2^ = 0.0%, *p* = 0.585), so a fixed-effect model was used. The results revealed that cachexia was significantly associated with worse PFS (HR = 1.84, 95% CI: 1.59–2.12, *p* < 0.001). The results were consistent with the above finding in univariate (HR = 1.90, 95% CI: 1.56–2.30, *p* < 0.001) and multivariate (HR = 1.76, 95% CI: 1.41–2.19, *p* < 0.001) analyses (Figure 5). Besides, we also found that patients with cachexia had considerably shorter TTF than those without cachexia (HR = 2.15, 95% CI: 1.32–3.50, *p* = 0.002, Supplementary Figure 2).

### Pre-immunotherapy cachexia and ORR

**Figure 6:**
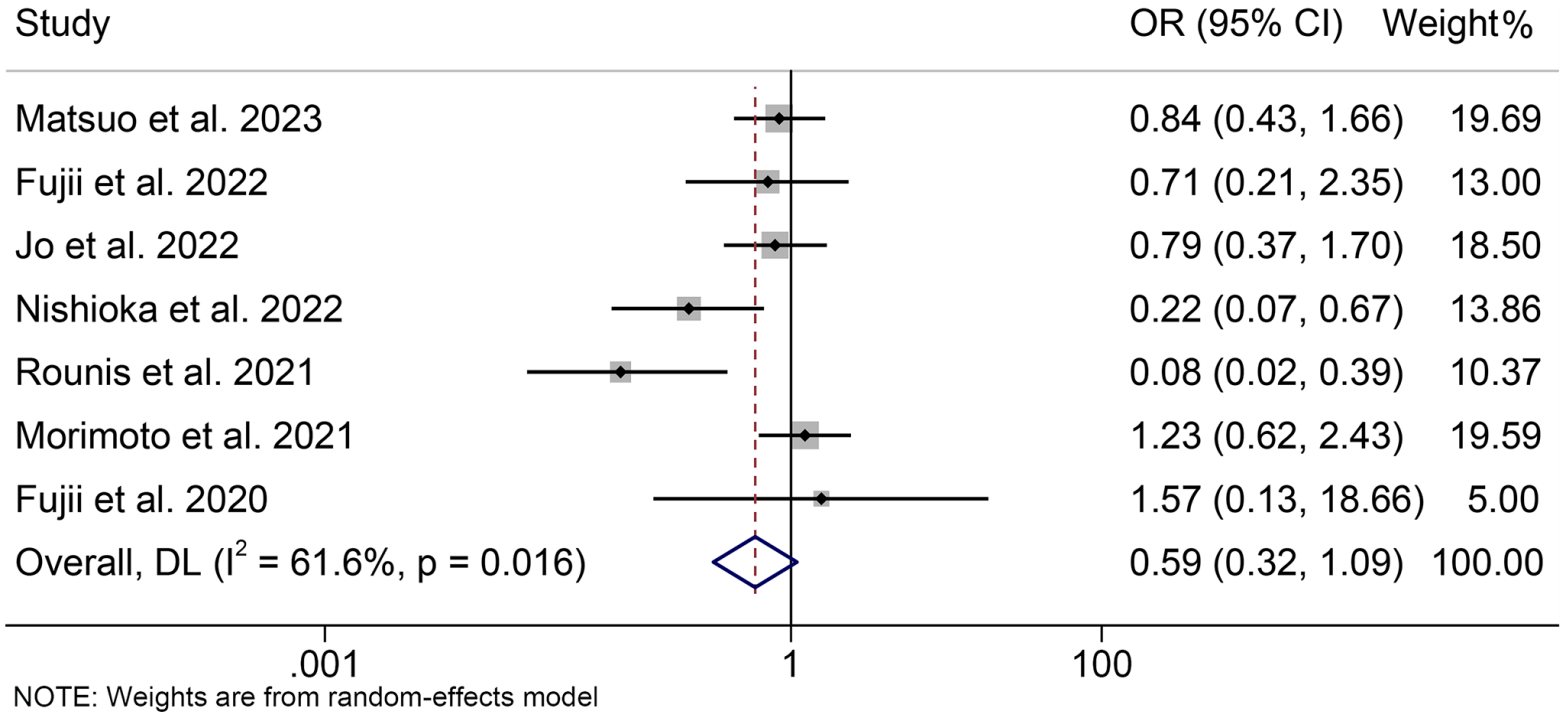
**Forest plots of the relationship between cachexia and objective response rate.** Abbreviations: OR: odds ratio; CI: confidence interval; DL: DerSimonian-Laird method.

We also assessed the relationship between cachexia and the overall response rate. Due to significant heterogeneity, we used a random effects model (I^2^ = 61.6%, *p* = 0.016). The pooled analysis indicated that there was a trend towards a lower ORR in cancer patients with cachexia compared to those without cachexia, although not statistically significant (OR = 0.59, 95% CI: 0.32–1.09, *p* = 0.093, Figure 6). Besides, pooled analysis that included only NSCLC patients was consistent with the above findings (OR = 0.55, 95% CI: 0.29–1.06, *p* = 0.076, Supplementary Figure 3).

### Sensitivity analysis and publication bias

**Figure 7:**
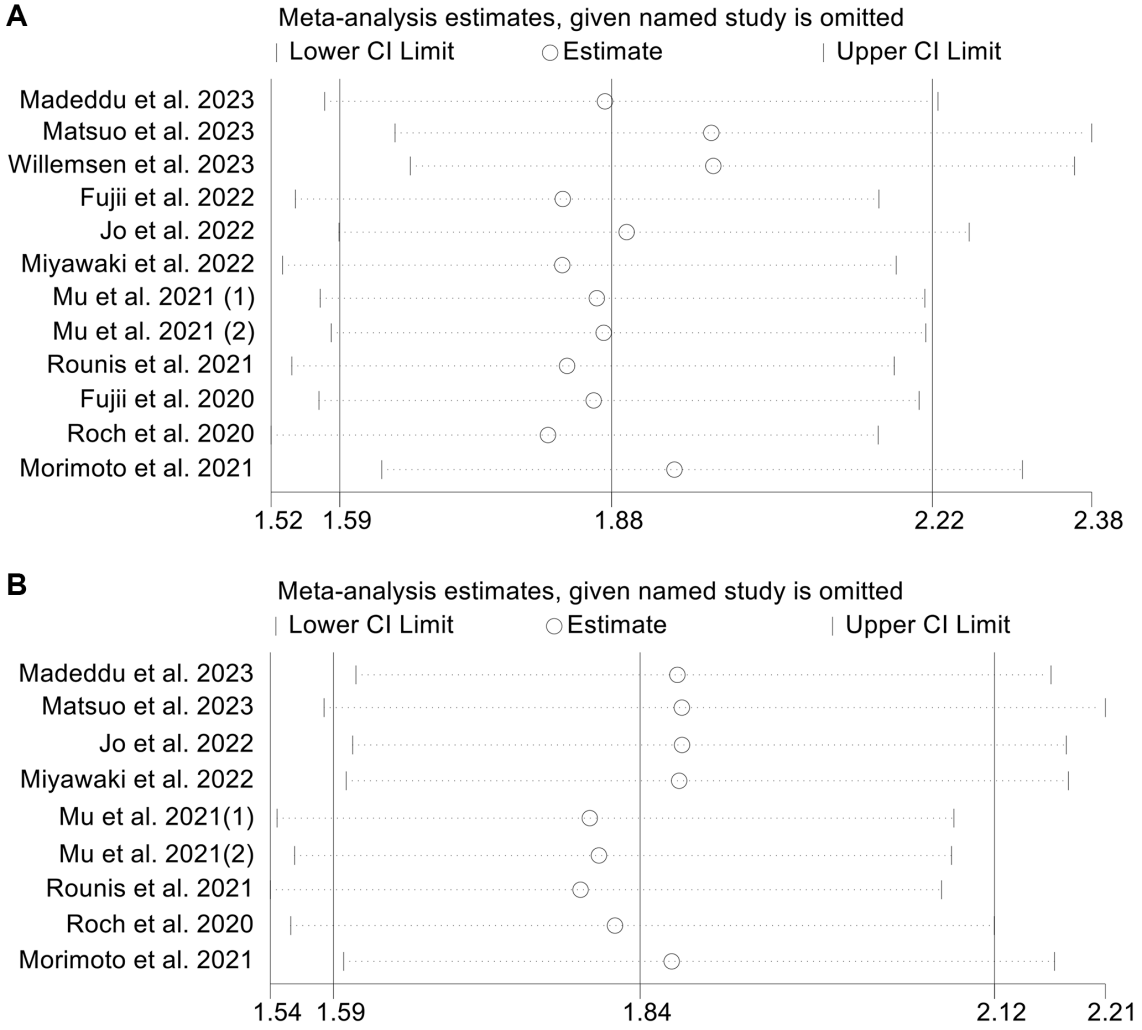
Sensitivity analysis of the association between cachexia and overall survival (**A**) and progression-free survival (**B**). Abbreviations: HR: hazard ratio; CI: confidence interval.

OS and PFS, the primary outcome indicators in this study, were subjected to sensitivity analyses and publication bias tests. The sensitivity analysis results are provided in Figure 7A, 7B. When any individual study was eliminated from the analysis, the pooled HRs for OS and PFS were similar. In addition, sensitivity analysis of ORR also confirmed that the above results are stable (Supplementary Figure 4). The funnel plot, Begg’s test (OS, *p* = 0.224; PFS, *p* = 0.602) and Egger’s test (OS, *p* = 0.077; PFS, *p* = 0.134) did not reveal any publication bias in OS (Supplementary Figure 5A) and PFS (Supplementary Figure 5B).

## DISCUSSION

With its outstanding efficacy and safety, immunotherapy using PD-(L)1 and CTLA-4 inhibitors has completely changed the way cancer patients are treated [33, 34]. However, it has been discovered that the therapeutic success of ICIs varies significantly among cancer patients, and there is still a lack of specific and reliable predictors of ICI efficacy. Cachexia, in particular, is frequent in cancer patients. This meta-analysis represents the first attempt, to our knowledge, to systematically assess the correlations between cachexia and the clinical outcomes of ICI-treated patients. The pooled data demonstrated that cachexia was significantly associated with a poorer OS and PFS.

The metabolic alterations linked to cachexia can reduce anti-tumor immunity. The release of pro-inflammatory cytokines, including TNF-α, IL-6, and IL-1, is provoked by cancer cells, which sets off a chain reaction that results in weight loss by causing the breakdown of skeletal muscle and adipose tissue as well as anorexia [12]. These factors also upregulate the release of corticotropin-releasing hormone while concurrently suppressing ghrelin, intensifying the loss of appetite [35]. Flint and colleagues demonstrated that tumor-induced IL-6 suppresses the production of hepatic ketone bodies, resulting in the significant secretion of glucocorticoids during periods of caloric deficiency [36]. Moreover, their research unveiled that this stress-induced hormonal response stifled immune activity within tumors, ultimately culminating in the failure of anticancer immunotherapy [36].

Cachexia may reduce the efficacy of ICIs in patients with NSCLC and high PD-L1 expression, according to a previous single-center retrospective analysis [37, 38]. This may be due to the fact that in NSCLC patients with cachexia, IL-6, IL-1, and TNF-α reduce CD8^+^ tumor infiltrating lymphocytes and anti-tumor immunity [37]. Studies have also confirmed that inhibition of cytokine pathways associated with cachexia formation has been shown to enhance the anti-cancer immune response [39, 40], and the combined blocking of specific cachexia-promoting mediators and the PD-1/PD-L1 axis has been shown to have a synergistic effect [41, 42]. Our conclusions are consistent with the above findings that cachexia shortens OS and PFS after cancer patients receiving ICI therapy.

In the context of cachexia management, a spectrum of therapeutic options, encompassing both pharmaceutical and non-pharmaceutical approaches, is available. Within the realm of pharmaceutical treatments, corticosteroids, non-steroidal anti-inflammatory drugs, and progesterone have demonstrated efficacy [43, 44]. It’s worth noting, however, that these treatments are accompanied by the risk of adverse events [43, 44]. Anamorelin, an orally administered, high-affinity, selective ghrelin receptor agonist, has exhibited the ability to significantly increase lean body mass in patients with advanced NSCLC, although it did not yield a significant improvement in handgrip strength [45]. Non-pharmacological interventions encompass dietary management [46] and structured physical exercise regimens [47]. Yet, physical exercise poses a challenge, as many advanced cancer patients tend to discontinue participation [48]. Additionally, interventions solely relying on either pharmaceutical or dietary approaches are less than fully effective [49]. Therefore, combining medication with diet management and physical exercise is an urgently needed holistic approach to improve ICI treatment outcomes for cancer patients with cachexia.

Some limitations of the present meta-analysis are to be addressed. First, most of the included studies were retrospective. Most of the included studies collected patient data retrospectively. The diagnostic criteria for cachexia were not entirely consistent between studies, although each study stated that their diagnostic criteria for cachexia referenced Fearon et al. [12]. Finally, although we found that cachexia was associated with a lower ORR, it was not statistically significant and more studies need to be included to explore the relationship.

## CONCLUSION

Cancer cachexia is related to poor clinical outcomes in ICI-treated cancer patients and may be beneficial in identifying therapy indications. Early intervention to improve cachexia is thought to be significant for ICI treatment success and should be considered in the future.

## Supplementary Materials

Supplementary Figures

Supplementary File 1
