# Supplementary material for: Cancer cachexia reduces the efficacy of immune checkpoint inhibitors in cancer patients
Source: Aging (Albany NY). 2024 Mar 11;16(6):5354–69. doi: 10.18632/aging.205652 (PMC11006492; doi:10.18632/aging.205652)
Supplement: Supplementary Figures [file aging-16-205652-s001.pdf]

SUPPLEMENTARY FIGURES

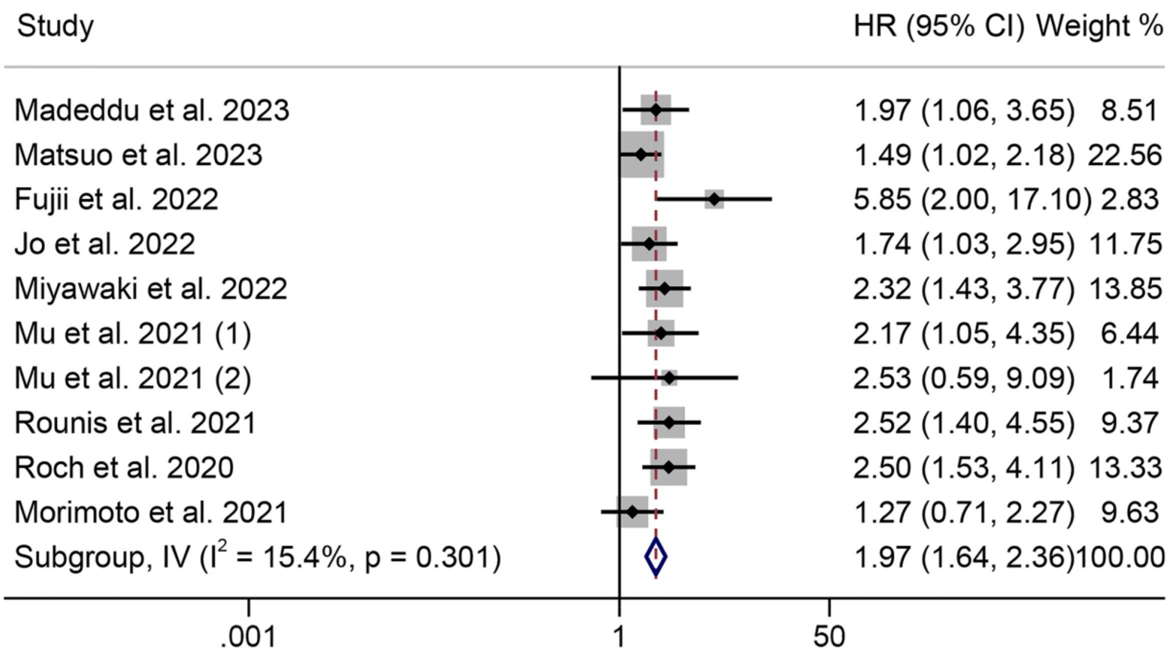

Supplementary Figure 1. Forest plots of the relationship between cachexia and overall survival in NSCLC patients. Abbreviations: HR: hazard ratio; CI: confidence interval; IV: Inverse Variance method.

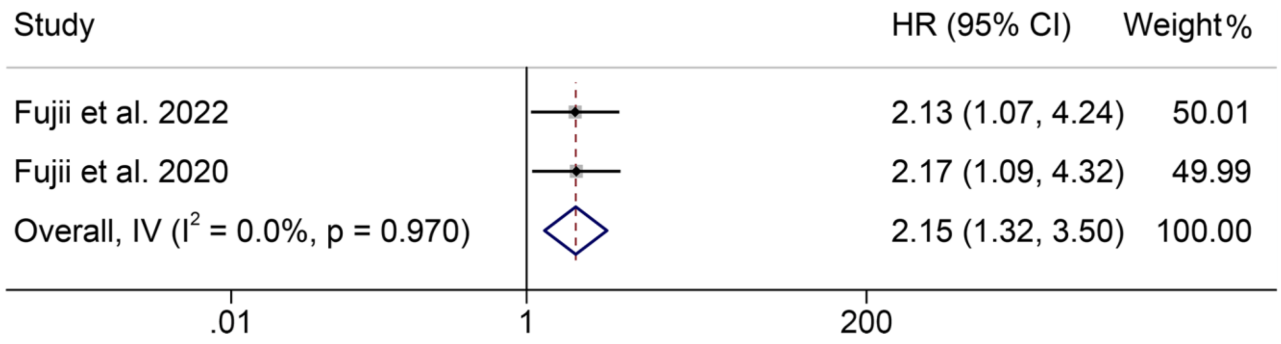

Supplementary Figure 2. Forest plots of the relationship between cachexia and time to treatment failure. Abbreviations: HR: hazard ratio; CI: confidence interval.

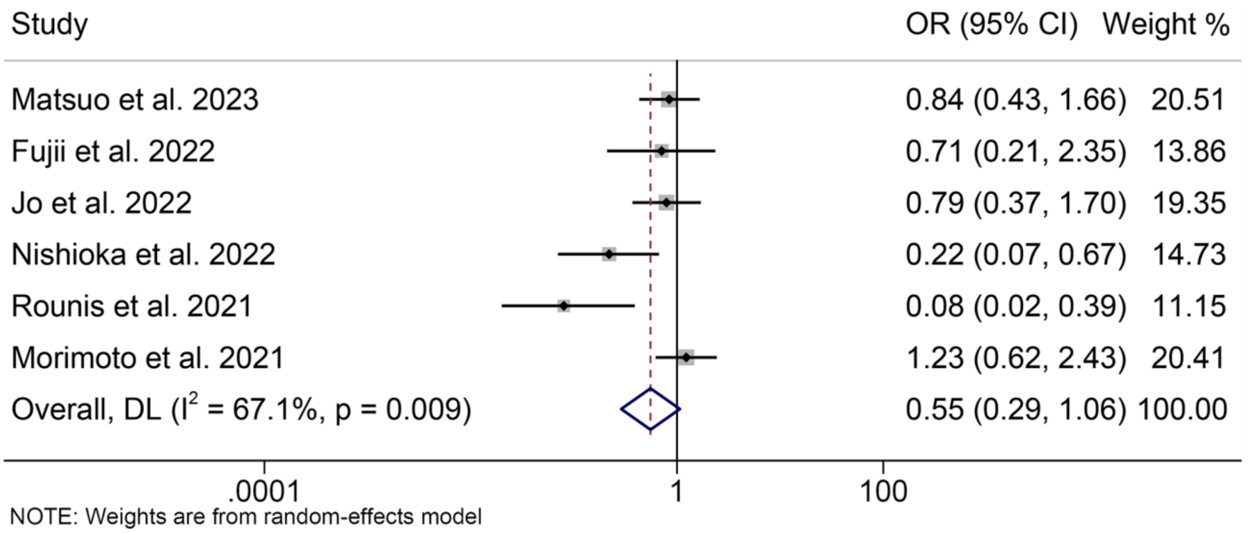

Supplementary Figure 3. Forest plots of the relationship between cachexia and objective response rate in NSCLC patients. Abbreviations: OR: odds ratio; CI: confidence interval; IV: Inverse Variance method.

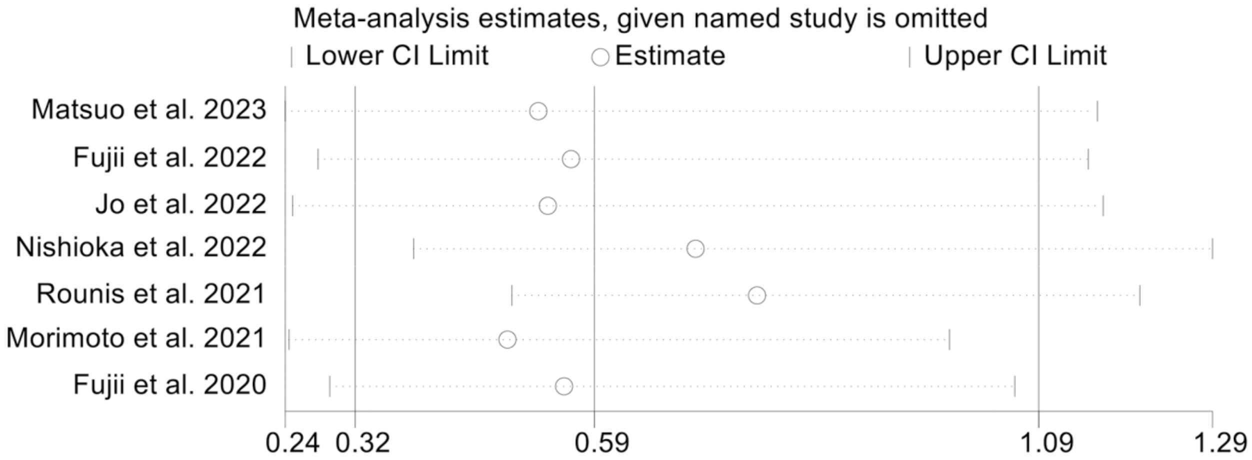

Supplementary Figure 4. Sensitivity analysis of the association between cachexia and objective response rate. Abbreviations: HR: hazard ratio; CI: confidence interval.

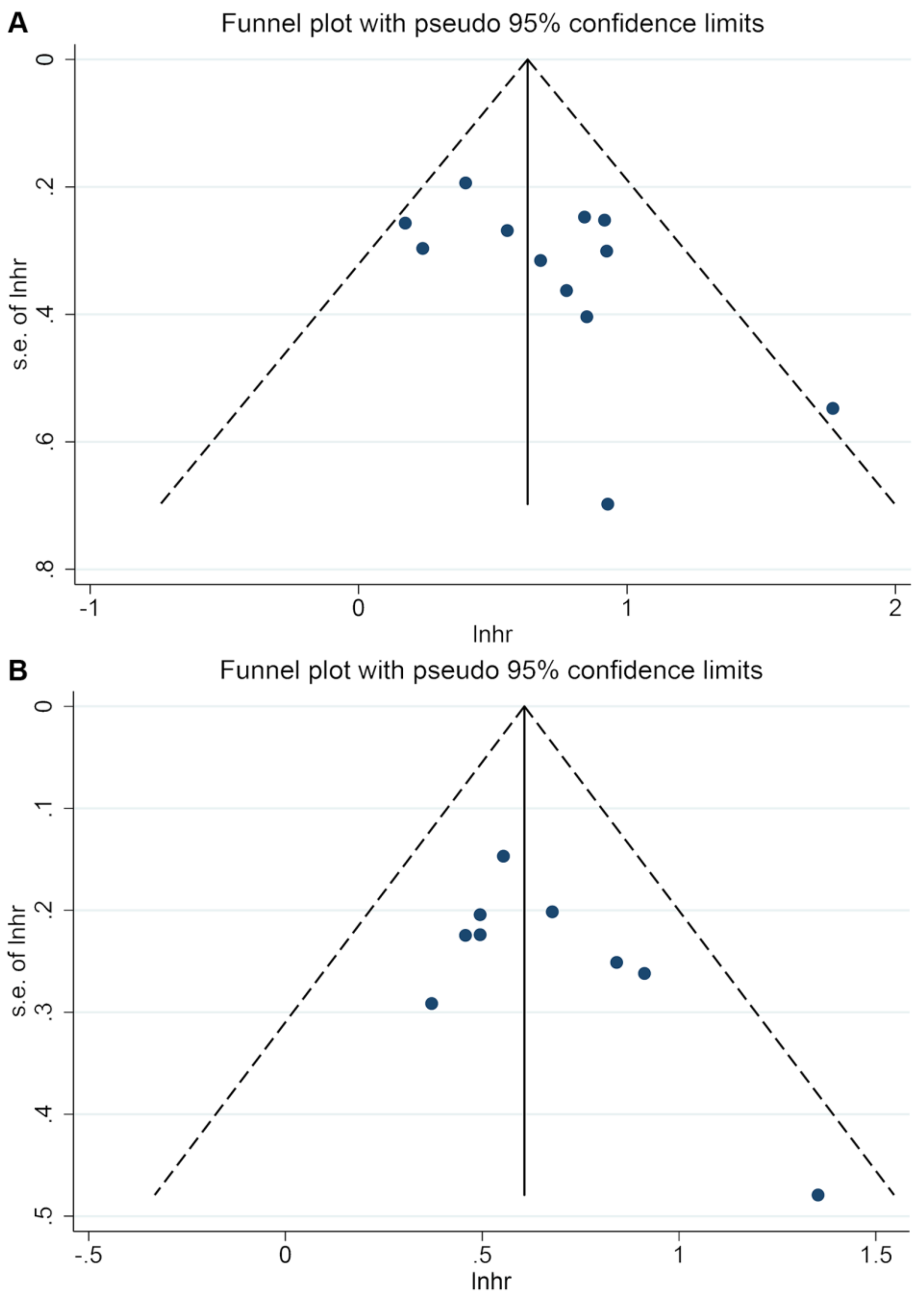

Supplementary Figure 5. Funnel plots of the relationship between cachexia and overall survival (**A**) and progression-free survival (**B**). Abbreviations: HR: hazard ratio; DL: DerSimonian-Laird method.
