# Supplementary material for: Cancer cachexia reduces the efficacy of immune checkpoint inhibitors in cancer patients
Source: Aging (Albany NY). 2024 Mar 11;16(6):5354–69. doi: 10.18632/aging.205652 (PMC11006492; doi:10.18632/aging.205652)
Supplement: Supplementary File 1 [file aging-16-205652-s002.pdf]

SUPPLEMENTARY FILE

Supplementary File 1. Detailed search strategy.

((Weight Reduction) OR (Weight Loss) OR (“Weight Loss”[Mesh]) OR (Cachexia) OR (“Cachexia”[Mesh])) AND (((Camrelizumab) OR (Sintilimab)) OR (Tislelizumab)) OR (Toripalimab)) OR (Envafolimab)) OR (Immune Checkpoint Inhibitors)) OR (Checkpoint Inhibitors, Immune)) OR (Immune Checkpoint Inhibitor)) OR (Checkpoint Inhibitor, Immune)) OR (Immune Checkpoint Blockers)) OR (Checkpoint Blockers, Immune)) OR (Immune Checkpoint Blockade)) OR (Checkpoint Blockade, Immune)) OR (Immune Checkpoint Inhibition)) OR (Checkpoint Inhibition, Immune)) OR (PD-L1 Inhibitors)) OR (PD L1 Inhibitors)) OR (PD-L1 Inhibitor)) OR (PD L1 Inhibitor)) OR (Programmed Death-Ligand 1 Inhibitors)) OR (Programmed Death Ligand 1 Inhibitors)) OR (PD-1-PD-L1 Blockade)) OR (Blockade, PD-1-PD-L1)) OR (PD 1 PD L1 Blockade)) OR (CTLA-4 Inhibitors)) OR (CTLA 4 Inhibitors)) OR (CTLA-4 Inhibitor)) OR (CTLA 4 Inhibitor)) OR (Cytotoxic T-Lymphocyte-Associated Protein 4 Inhibitors)) OR (Cytotoxic T Lymphocyte Associated Protein 4 Inhibitors)) OR (Cytotoxic T-Lymphocyte-Associated Protein 4 Inhibitor)) OR (Cytotoxic T Lymphocyte Associated Protein 4 Inhibitor)) OR (PD-1 Inhibitors)) OR (PD-1 Inhibitor)) OR (PD 1 Inhibitors)) OR (Inhibitor, PD-1)) OR (PD 1 Inhibitor)) OR (Programmed Cell Death Protein 1 Inhibitor)) OR (Programmed Cell Death Protein 1 Inhibitors)) OR (Pembrolizumab)) OR (Nivolumab)) OR (Atezolizumab)) OR (Ipilimumab)) OR (Avelumab)) OR (Tremelimumab)) OR (Durvalumab)) OR (Cemiplimab)) OR (Immune Checkpoint Inhibitors[MeSH Terms])).
